# Medical Society of the County of Albany

**Published:** 1873-12

**Authors:** F. C. Curtis

**Affiliations:** Secretary


ART. IV.—*Medical Society of the County of Albany.* Annual Meeting, November 11th, 1873.

Reported by F. C. CURTIS, M. D., Secretary.

The annual meeting of the Albany County Medical Society was held at the City Hall, in Albany, Dr. ALBERT VAN DERVEER, President, in the chair. There were present about seventy members. After the minutes of the last semi-annual meeting had been read and approved, the Secretary presented a communication from the Committee on Hygiene of the State Society which after setting forth the character and duties of this committee, requested the appointment of a similar committee in this society, and that it report to them answers to certain questions regarding the subject of Drainage in this county. On motion the communication was received and adopted, and a committee appointed consisting of Drs. LEVI MOORE, S. H. FREEMAN and J. M. BIGELOW.

The President called the attention of the Society to "A Memorial of the American Medical Association with regard to the rank of the Medical Corps of the U. S. Army.

Dr. J. P. BOYD, chairman of the board of censors, reported favorably upon Drs C. E. Segar, P. E. Feuelly and O. H. E. Clarke, applicants for membership, and on motion they were elected members.

Dr. W. H. MURRAY, treasurer, reported the society to be financially in a flourishing condition.

The report of the committee on publishing, the second volume of the Annals of the Society being called for, Dr. JAMES S. BAILEY, reported that 500 copies had been published, 239 subscribed for, of which number 102 copies had not been taken; proceeds of sales amounted to $411, leaving $482 still due the publisher. A protracted debate upon the report took place, and it was finally received and laid upon the table for discussion at another time.

The names of Dr. O. D. BALL, Dr. L. BOUDRIAS, and Dr. MARY DU BOIS, were proposed for membership and referred to the Board of Censors.

The President, Dr. VAN DERVEER, then delivered the Annual Address as follows:

Gentlemen of the Society: Our gathering to-day reminds us that as a Society we have come to another milestone. I am reminded that to-day I give back the trust committed to me at this year's beginning.

While grateful for the honor you then conferred upon me, I was, at the same time, conscious of inability and inexperience. The only merit I felt I could claim, was an earnest desire for the advancement of our Society, and a willingness to devote to this end the best efforts of my head and heart. Prompted by this, and the hope that I would receive efficient aid from you I entered upon my work. This hope has never failed me, and for the charity you have thrown over my failings, and the help you have given me I can only offer the simple words, "I thank you." You will find your reward in the condition of our Society. Numerically we are strong. New members have come to us during the year, and at present there are but two county medical societies in the State that excel us in numbers. As you observe from the report of the treasurer, financially our condition is good. Our record for the past year is one to which we can look back with feelings of gratification. We have had more than our usual number of meetings, and I think you will all agree with me that the most of them were both pleasant and profitable. In December a proposition was made to give an entertainment to the State Society. This was readily acceded to, and the funds to provide for such entertainment were promptly and liberally subscribed The result was a pleasant gathering from which mutual benefit and pleasure were derived.

But mingling with our congratulations at the success of the past year, there is a note of sadness as we think of those we have lost by death. We miss to-day Dr. John P. Witbeck, Dr. Uriah G. Bigelow and Dr. John H. Becker, men whose record is might in their respective fields of practice, and whose noble work is cherished not only by us but by many loved patients who hold them in grateful remembrance. Appropriate tributes of respect to their memory have already been paid by us as a Society. As individuals let us emulate their virtues, profiting by the good they have done.

I would call your attention to one great want of the Society, and that is the necessity of securing a proper room in which its meetings can be held. It is not needful that I should dwell at length upon this. You must all have experienced the inconvenience to which we are subject from the present arrangement. The benefit accruing from a room containing a selection of late books and medical journals to which the members could at any time have access must be obvious to all. I would urge that efforts be made to secure this at as early a day as is possible.

And now, for a few minutes, I would call your attention to the subject I have selected for my annual address. I present you no learned dissertation on the science of medicine or surgery. It is enough to know that rapid advances are being made in the different specialties that go to make up our noble profession, that errors are being rooted out and the vacant spaces filled by facts that statistics help us so surely and promply to establish. We can say with all truthfulness that great credit is due to that noble class of men who, taking up a single line of thought and observation, have helped to place us as a profession beyond the pen of sarcasm or the sting of reproach. Firm must be the principles and theories of medicine and surgery that can stand the close observation and careful investigation of the medical thinkers and writers of the present time.

The address was continued with a paper on the subject of *Stricture of the Urethra.* The anatomy of the tissues involved was briefly sketched and then the clinical history of stricture was detailed. The different forms of stricture were spoken of, such as idiopathic and traumatic, strictures of large and small calibre, short, continuous, spiral and tortuous strictures, and the diagnostic points of each. More particularly the means of detection of strictures, as well as their location and characters, with bulbous and olive pointed bougies were discussed. Finally the methods of treatment by dilitation, by divulsion, and by internal urethrotomy were presented, and a very complete set of the various instruments used in carrying out these several treatments were shown, including among others Gouley's tunnelled instruments, the value of which was spoken highly of.

A large number of cases of stricture which had fallen under his treatment were given by Dr. Van Derveer, illustrating various form of the affection and the different methods of treatment.*

The following case illustrates the benefit resulting from gradual dilatation in the treatment of obstinate gleet due to incipient stricture.

April 10th, 1873, W. J., but 22; habits temperate. Has had two attacks of gonorrhœa, the last a year ago, since which time there has been a constant gleety discharge. Twice after severe exposure has been obliged to resort to the use of the catheter to draw off his urine, the instrument being introduced each time with little trouble. Examination with bulbous bougie reveals a slight stricture just back of the fossa navicularies, the remaining portion of the urethra appearing of normal calibre. Nos. 13 and 15 steel sounds were passed twice a week for a month, when the discharge entirely ceased, and has not returned up to the time when last seen in August,

* This collection of cases is to be published elsewhere. else I should be glad to present some of them here. This single short one is given, being a duplicate of another, not because it is in any way a specimen of the rest.

1873. His general health being good no medicine was given internally.

The paper concluded with a summing up of the facts deduced from the cases and their treatment.

Stricture situated in the membranous part, and bulbous portion of the spongy part of the urethra are best treated where it is possible by gradual dilatation; if this is not possible, by divulsion. For stricture of the spongy part internal urethrotomy is preferable, being much less painful than gradual dilatation here. The use of the meatotome was thought to be allowable where the dilating instrument in performing gradual dilatation causes great pain.

At the conclusion of the President's address, Dr. Quackenbush moved that a vote of thanks be tendered the President for his able and entertaining address. The motion was adopted.

Dr. Hailes moved that a copy of the President's address be requested for publication in the Transactions of the State Society. The motion was adopted.

The election of officers for the ensuing year being next in order, Dr. JAMES S. BAILEY, whose name had been proposed for President, nominated for that position Dr. JOHN W. SWINBURNE. In thus declining the office himself he said that it was with no lack of appreciation of the kind interest which his friends had taken in him, nor from a fear of their inability to elect him. But he did it for the good of the society, and to promote harmony, since he believed Dr. Swinburne could not fail to be acceptable to all, and his election would best further the interest and welfare of the Society. Moreover his age, his position in the profession which he had attained unaided and his reputation which was not merely a local one, entitled him to the position. His first choice was Dr. Swinburne; but, owing to pressing engagements, Dr. Swinburne stated that he would be unable to accept the office. Since that time, however, circumstances had transpired which would make it possible for him to accept it. He, therefore, willingly withdrew in his favor.

The following officers were then elected, unanimously, for the ensuing year:

*President*—John Swinburne.

*Vice-President*—H. W. Steenbergh.

*Secretary*—F. C. Curtis.

*Treasurer*—W. H. T. Reynolds.

The remainder of the officers coutinue the same as last year.

On motion of Dr. Craig, the Society took a recess to Congress Hall.

The Society met again at Congress Hall at the close of the business session of the annual meeting, to celebrate the fiftieth anniversary in the practice of medicine of Dr. P. P. STAATS, and Dr. B. B. FREDENBURGH.

The President, Dr. Van Derveer, addressed the Society in the following words:

It is our good fortune at this time, to participate in the pleasures of an occasion, as gratifying as it is rare.

The custom of our Society, in obedience to which we have assembled, is one well honored in its observance.

Surely, if there are any whose years of toil and devotion merit recognition at the hands of their co-laborers, the physician, who through half a century has been faithful to the call of duty, even though it led to danger, is one who should thus be honored.

I am happy to welcome, as our guests to-night, two who have been thus found faithful. Drs. P. P. Staats and B. B. Fredenburgh, are too well known in our Society, have been too long identified with its interests, to need other introduction than the mere mentioning of their names.

Fifty years! To us who are in the morning of our profession, how the lines lengthen, as we attempt to look out fifty years in the future. To our venerable associates, as they look back, the lines draw nearer and nearer, until the point of meeting seems so close that with outstretched arm, they can rest their hands upon it. But what breadth of experience do these lines eontain. Could our friends recount to us, the hopes, the fears, the joys, the disappointments, the struggles, the hours of anxious waiting and watching, the nights of study, that go to make up these fifty years, we would shrink from such experience.

But they would tell us, that with all these came some bright days—days when their patient, persistent labors were rewarded—and they listened to the words which grateful hearts uttered, when homes over which the death angel seemed hovering, were filled with joy as the loved one in convalesence came back to life and health.

To you, our fathers in our profession, we would now tender an earnest welcome. We rejoice to be able to show you that we appreciate your labors.

If the occasion which commemorates fifty years of domestic life, is called a golden wedding, the year which commemorates fifty years of active service in so arduous a profession should be called a golden year. May you so find it. Though the mist of doubt may have enshrouded your morning, at times have overshadowed your noon-day, we trust you will both realize the fulfillment of the promise that, "At evening time it shall be light."

At the conclusion of Dr. Van Derveer's remarks, Dr. JAS. McNAUGHTON, chairman of the entertainment committee, made a brief and characteristic address to Drs. STAATS and FREDENBURGH, the gentlemen in whose honor the banquet was given. Dr. P. P STAATS responded as follows:

*Mr. President and Members of this Society:*

Gentlemen—It affords me great pleasure to meet you here on this occasion, particularly as it manifests your appreciation of my long service in the profession. I am the more grateful, knowing that I have done very little to deserve the compliment you have paid me this evening. It is another evidence of the kindness and courtesy I have always received from you. Please accept my sincere thanks for the past, and an earnest desire for its continuance in the future.

On the 22d of February, 1823, I received my diploma from the Medical Society, in the city of New York. The censors for the southern district of this State were Felix Pascalis, James R. Manly and Charles Drake. At that time it was expensive to attend two full courses of lectures, which was then, as now, required to enable a student to graduate at a medical college. It was more expensive than many could afford, and the law of the State made it only honorary, requiring a license in order to practice. The intrinsic merit had very little to do with the M. D., which law, requiring another license, has since been repealed. The examination before the State Medical Society was full as thorough and rigid as it was before the Professors of the Medical College. In fact, I was cognizant of an instance when the State Censors rejected a student, who on appeal to the Medical College, passed and graduated. The law at present, allowing any one to practice medicine in this State, is wrong, and should be amended or repealed. There is no protection for the public against gross imposition. Strangers, sojourneying in our state, from other States, where the law provides against such imposition, requiring a guarantee of his qualifications before he is allowed to practice, are not aware of the liberty the State of New York gives to such a fraud, until it is too late to remedy the evil. On the 1st of March, 1823, I commenced the practice of medicine and surgery in partnership with my brother, the late Barent P. Staats in this city. At that time, the only hospital there was in this city was the alms-house, where all subjects in destitution were cared for, and at that time the Erie canal was in course of construction, and it, too, contributed largely in supplying that institution with patients, This necessarily furnished a large field for the practice of medicine and surgery, and as my brother, who held the appointment of County Physician (there were no City Physicians at that time), so that all came under the charge and care of the County Physician, and as I was the junior partner, I had to take charge of the medical and surgical department of that institution, which gave me a good opportunity to attend to an extensive practice. In fact, I soon became familiar with almost every disease, and soon became self-reliant; for in cases of emergency there could be no delay, and the only course was to take the responsibility and discharge my duty, which I faithfully endeavored to. Our medical profession then consisted of the following gentlemen, viz.: Elias Willard, William Bay, Jonathan Eights, Charles D. Townsend, Platt Williams, Joel A. Wing, Peter Wendell, James McNaughton, Barent P. Staats, John James, Alden March, Peter Van Olinda, S. Treat, and some younger, who I do not now remember; gentlemen who gave their time and services to the profession, and were ornaments to it; who were governed in their judgments by experience in practice, and were not carried away by romance or sophistry in theory. It is true that great advancement has been made in our profession during the last fifty years, but it is also true that if a more rigid scrutiny had been observed by our profession and the press to weed out many errors which now are allowed to occupy a place they do not deserve, by the indorsement of our societies, without a thorough investigation of their merits, the professional standard would have attained a more enviable position than it even now does. I did not become a member of this Society until the law of the State, in 1828, made it incumbant upon all practitioners to join the Society, for the reason that occasionally then, as since, some personal feelings would be carried into the Society meetings, which would produce discord in the Society, and unpleasant feelings among its members would be the result; and, as I am no advocate for such a course, I preferred to remain an outsider rather than partake in any of those discords; consequently, my advancement to the honors of the Society have not been so rapid as many others my juniors; still, I have received all the honors I desire, and it has been through the courtesy of the Society, without ever making myself a candidate for any office in the Society. I have had the degree of M. D. conferred upon me by the State Medical Society. I have been elected a delegate from this to the State Medical Society. I was elected unanimously President of this Society, and at the expiration of my term, four years as a delegate from this Society. I was elected a permanent member of the State Medical Society, and now last, though not least, I am complimented by having my fiftieth anniversary of medical practice celebrated by this Society. Gentlemen, I have had opportunity to witness the different theories of pathology from the first solids, second fluids, and third and last, now the cells, as the exciting cause of all abnormal difficulties. I think there is more advantage gained for the patient to have a correct diagnosis made by his physician, and suitable therapeutic remedies administered, than to wait a post mortem development to decide the pathology of his disease.

Dr. B. B. FREDENBURGH, also reponded, as follows:

Mr. President—It affords me much pleasure to meet you here this evening. Poor health should have excused my presence, but having more friends than I was aware of I could not decline so many invitations. You have conferred this honor upon me which

I fully appreciate, and for which I tender you my thanks. The most of you I have not the pleasure of a personal acquaintance with, not having met with the society recently. The senior portion who I have met here have passed away and entered upon their rest, which forcibly reminds us that we too are mortal. I congratulate you on the success of the society, and hope that it may continue to prosper, and that you may each live to be the recipients of this honor, and so live, that when that inevitable hour arrives, you may look back upon duty well performed, upon temptations to achieve success by questionable means, firmly resisted, and

> "Sustained and soothed
> By an unfaltering trust, approach the grave
> Like one who wraps the drapery of his couch
> About him, and lies down to pleasant dreams."

At the close of the entertainment provided for the occasion, Dr. Staats offered the following sentiment, "The Young Men of the Profession," and called upon Dr. QUACKENBUSH to respond who, after making a few introductory remarks, recited an original poem of some length, which was received with applause. Other toasts and responses were then made and at ten o'clock the assemblage dispersed.

